# Early weaning from oxygen therapy in African children with severe pneumonia

**DOI:** 10.1186/s12916-025-04178-9

**Published:** 2025-07-01

**Authors:** Kathryn Maitland, Elisa Giallongo, Mainga Hamaluba, Florence Alaroker, Robert O. Opoka, Abner Tagoola, Damalie Nalwanga, Eva Nabawanuka, William Okiror, Margaret Nakuya, Denis Aromut, Thomas N. Williams, Karen Thomas, David A. Harrison, Paul Mouncey, Andrew Bush, J. F. Fraser, Kathy Rowan, Peter Olupot-Olupot, Sarah Kiguli, for the COAST trial group

**Affiliations:** 1 Department Surgery and Cancer, Institute of Global Health Innovation, Imperial College London, London, UK; 2 Kenya Medical Research Institute (KEMRI) Wellcome Trust Research Programme, Kilifi, Kenya; 3 Clinical Trials Unit, Intensive Care National Audit & Research Centre (ICNARC), London, UK; 4 Soroti Regional Referral Hospital, Soroti, Uganda; 5 Department of Paediatrics, School of Medicine, Makerere University and Mulago Hospital, Kampala, Uganda; 6 Jinja Regional Referral Hospital, Jinja, Uganda; 7 Faculty of Health Sciences, Busitema University, Mbale Campus, Mbale, Uganda; 8 Mbale Clinical Research Institute, Mbale, Uganda; 9 Department of Radiology, School of Medicine, Makerere University, Kampala, Uganda; 10 Imperial Centre for Paediatrics and Child Health, St Marys Hospital, London, UK; 11 Department of Paediatric Respiratory Medicine, Royal Brompton Hospital, London, UK; 12 Critical Care Research Group and Intensive Care Service, University of Queenslandand, The Prince Charles Hospital , Brisbane, Australia

**Keywords:** Severe pneumonia, African children, Oxygen therapy, High flow nasal therapy

## Abstract

**Background:**

In Africa, severe pneumonia remains the major cause of paediatric hospitalisation, resulting in high requirements for oxygen therapy. Adequate supplies of oxygen are key challenges for many low-resource hospitals. The World Health Organization manual for oxygen therapy advises 2–3 days of oxygen therapy for pneumonia and recommends against early weaning, even in the absence of hypoxaemia. Few data support this recommendation. We describe the oxygen use and timing of weaning in the COAST trial of oxygen therapy (ISRCTN15622505).

**Methods:**

Children aged 28 days to 12 years presenting to 6 hospitals in Uganda and Kenya with severe pneumonia and hypoxaemia (saturations < 92% on pulse oximetry (SpO_2_) were eligible for the trial. Children in two strata (a) severe hypoxaemia (SpO_2_ < 80%) and (b) moderate hypoxaemia (SpO_2_ 80–91%) were allocated to receive high flow nasal therapy (HFNT), low flow oxygen delivery (LFO) or control (no immediate oxygen (moderate hypoxaemia stratum only)). Children were closely monitored over 48 h by pulse oximetry and weaned off oxygen once SpO_2_ > 92%. We describe the oxygen use and proportion requiring respiratory support over time by intervention strategy.

**Results:**

Of the 1842 children enroled the majority, 1454 (79%) had moderate hypoxaemia. In this stratum, by 2 and 8 h, 148 (41%) and 200/360 (55.6%) in the LFO arm had been weaned; in the HFNT arm, 213/362 (59%) were receiving respiratory support at 2 h in room alone, and by 8 h, 164/362 (45%) had been weaned. At 48 h, in the respective strata, 77–80% and 53–63% still had respiratory distress but without hypoxaemia and were thus not receiving oxygen. Median oxygen use at 48 h in the moderate hypoxaemia group was highest in LFO am 480L (IQR 236.2, 2132.2) compared to 113.4 L (IQR 0.0, 1453.9) in the HFNT and 0 L (IQR 0.0) in the control arms. Children requiring oxygen beyond 48 h, 17/33 (51.1%) and 9/46 (19.5%) in the respective strata, had additional cardiac conditions.

**Conclusions:**

Closely monitoring SpO_2_ resulted in early weaning and reduced the use of and exposure to oxygen. Where oxygen supplies are at a premium, this approach may improve equitable access for children with severe pneumonia.

**Supplementary Information:**

The online version contains supplementary material available at 10.1186/s12916-025-04178-9.

## Background

Pneumonia remains the leading cause of childhood death in sub-Saharan Africa [1, 2]. For children hospitalised with severe pneumonia, the World Health Organization (WHO) recommends oxygen therapy for those with clinical signs and symptoms of severe or very severe pneumonia. When pulse oximetry is available, the recommendation includes those with hypoxaemia (oxygen saturation (SpO_2_) of < 90) [3]. Whilst oxygen has been on the WHO Essential Medicines List for anaesthesia since the early 2000s, hypoxaemia was only added as an indication for oxygen in 2017 [4]. Similar changes in recommendations have also been added for children [5]. Nevertheless, oxygen supplies are often limited in sub-Saharan Africa and are difficult to maintain due to erratic supply chains, high costs, and dependence on unreliable electricity supplies [6–9]. The issue of ‘oxygen insecurity’ was highlighted in the 2022 Lancet Commission on Medical Oxygen Provision [10]. A scoping review of oxygen availability, published in 2021, reported wide variation across facilities (42–94%) [11]. Where oxygen was available, health care workers were reported to be ill-equipped to identify patients with hypoxaemia, to provide oxygen to those who needed it, or to know when to discontinue oxygen when appropriate. Finally, few hospitals have access to pulse oximeters [12]. Instead, as recently highlighted in a multicentre study from Kenya, there is a current reliance on non-specific clinical signs to guide oxygen therapy, which results in the poorly targeted use of a scarce and costly intervention [13]. The study also reported that even when functional pulse oximeters were available, 87% of the prescriptions for oxygen therapy were either unsupported by a pulse oximetry reading or were given to children with SpO2 > 90% [14]. Not only does the poor targeting of oxygen therapy increase demand and costs, but it also exposes children to the potential harms of unwarranted oxygen therapy [15]. The WHO manual for oxygen therapy indicates that in cases of severe pneumonia, hypoxaemia can last several days (usual duration 2–5 days) [2], and that correction of hypoxaemia should not guide oxygen weaning in a child with persistent emergency signs. It also recommends that weaning from oxygen should only be attempted once each day, preferably early in the morning. Few data are available, however, to support these recommendations.

The Children’s Oxygen Administration Strategies Trial (COAST) was designed [16] to address key research gaps for oxygen therapy identified by the WHO. This includes the conduct of randomised controlled trials to assess the benefit of oxygen therapy in children with hypoxaemia (oxygen saturation is < 90%) and to assess whether non-invasive respiratory support might improve outcome compared to standard oxygen delivery [17]. The main outcomes of COAST regarding efficacy have been reported elsewhere [18]. During the operationalisation of the trial, we revisited the evidence supporting current guidance on oxygen therapy (including weaning) and found limited data to support the current recommendations. Given the paucity of published data on this topic, we present granular details on the methods of oxygen delivery we adapted for the trial that included novel and innovative strategies to provide respiratory support with high-flow nasal therapy (HFNT) with minimal use of oxygen and early weaning for all non-hypoxaemic participants.

## Methods

Detailed methods for the COAST trial have been published, which are briefly summarised here. The COAST trial enroled children aged 1 month to 12 years who screened positive at the point of hospital admission for clinical signs and symptoms of severe pneumonia and hypoxaemia (an SpO_2_ of < 92% by pulse oximetry). The trial was conducted at 5 sites in Uganda and 1 hospital in Kenya. The open label randomised trial had two strata. Stratum A included children with clinical features of severe pneumonia and severe hypoxaemia (SpO_2_ < 80%). Stratum B included children with severe pneumonia who had moderate hypoxaemia (SpO_2_ 80–91%). The trial strata were identified using a logistic model examining the relationship between baseline (admission) oxygen saturation and mortality by 48 h in two datasets (details in Additional File: Background Figures S1, S2 and Table S1). This demonstrated that in children with respiratory features of pneumonia and SpO_2_ < 80%, 48-h mortality was 26–20%. For children with a baseline SpO_2_ between 80 and 91% 48-h mortality was 12% with no differences across the whole range. Children eligible for Stratum A (severe hypoxaemia (SpO_2_ < 80%)) were randomised to high flow nasal therapy (HFNT) via OptiFlow™ (experimental arm) or low flow oxygen (LFO) (standard of care arm per guideline) as a control group was not justifiable. Children eligible for Stratum B(moderate hypoxaemia (SpO_2_ 80–91%)) were randomised to HFNT via OptiFlow,™ LFO, or permissive hypoxaemia (control) to examine whether immediate receipt of oxygen improved outcomes. LFO was initiated in children in the control arm if their SpO_2_ recordings fell to < 80%. Both strata investigated whether HFNT led to superior outcomes compared to LFO (standard mode of oxygen delivery. BITMOS sat 801 + pulse oximeters (Bitmos GmbH, Sat 801 + , www.Bitmos.de) with paediatric probes were used for the measurement of oxygen saturations to assess study eligibility and to guide oxygen weaning. Weaning was guided by achieving correction of hypoxaemia (defined as SpO_2_ was ≥ 92% in our study) irrespective of whether the child had severity features (including respiratory distress [2]. Children were managed on general paediatric wards in facilities where mechanical ventilation facilities for children were not available. Children were reviewed at 15, 30, and 45 min and then at 1, 2, 4, 8, 16, 24, 36, and 48 h post enrolment. At each review, details of oxygen administration were recorded, and the percentage oxygen concentration delivered to the child (fraction of inspired oxygen or FiO_2_) was measured using a Handheld Oxygen Analyser VN202 MK11 with the sensor placed in the outflow. Actively solicited safety endpoints included suspected aspiration events, trauma from the nasal cannulae, and inability to tolerate high flow nasal therapy.

### Oxygen delivery methods and respiratory support

#### High flow nasal therapy (HFNT)

HFNT was delivered by Optiflow™ (Fisher and Paykel Healthcare, Auckland, New Zealand), which includes a humidifier with an integrated flow generator that delivers a high flow of warmed and humidified air/oxygen blend to spontaneously breathing patients. The blending of room air and oxygen thus permitted the trial to deliver respiratory support on room air alone (FiO_2_ 21%) or blended with minimal oxygen. The appropriate patient interface, breathing circuit, and OptiFlow settings were selected based on the manufacturers’ guidance. For children up to 12 kg, a paediatric circuit was used together with either an infant or paediatric patient interface. For children over 12 kg, adult circuits and small nasal cannula were used.

#### Optiflow flow rates

The Optiflow™ was set to junior mode for children ≤ 12 kg and adult mode otherwise. HFNT flow rates were 2 L/kg per minute for children 3–12 kg and were titrated to weight band for children > 12 kg (13–15 kg 30 L/min and 16–30 kg 35 L/min), as recommended by the manufacturer (Additional File Table S2). Details of the starting oxygen therapy (initiation), the changes required due to O_2_ saturation findings at the monitoring timepoint (titration) and stopping oxygen (weaning) were covered in a detailed manual of operations (Additional File Tables S3a–c). Children were ventilated with room air (21% O_2_) during the initiation period, with SpO_2_ monitored by pulse oximetry every 15 min in the first hour, with titrating additional oxygen (up to a maximum of 40%) with the aim of achieving an SpO_2_ ≥ 92%. A maximum FiO_2_ of 40% was permitted for only 30 min before down titration to 35% thereafter. Children who were unable to tolerate HFNT per-protocol were switched to receive LFO. Children were eligible for weaning after a minimum of 2 h of HFNT for those with oxygen saturations of ≥ 92%. Among those receiving additional oxygen, the aim was to initially titrate down the oxygen to room air alone while maintaining the flow rates. At 48 h following randomisation, any children still on HFNT and requiring additional oxygen were switched to LFO (standard mode of oxygen delivery).

#### Low flow oxygen

The method of delivery for LFO depended on local preference, but generally included a short nasal prong, catheter, or mask (plus a non-rebreathing reservoir for initial resuscitation). Infants were initiated on a flow rate of 1 L/min and children > 1 year on 2 L/min O_2_. Flow rates were then titrated up against oxygen saturation over the first 30 min to 1 h to a maximum of 2 L/min in infants and 4 L/min in older children, with the aim of achieving an SpO_2_ of ≥ 92%. If higher rates were required, children were switched to a mask and, depending on the type of mask, oxygen flows were gradually increased to 5 L/min, titrated against SpO_2_ measurements. Children were eligible for weaning after a minimum of 2 h over a period of 30 min if SpO_2_ was ≥ 92%. Monitoring was continued on weaned children, and LFO was restarted if SpO_2_ fell to < 92% before 48 h (Additional File Table S4). Crossover to HFNT was not permitted in the protocol.

#### Control (permissive hypoxaemia)

Children in the control arm were monitored in accordance with the trial protocol, and LFO therapy was initiated if their SpO_2_ (recorded over 5 min) fell to < 80%. Such children continued in the trial as described by the LFO protocol (above). Additional monitoring was permitted, driven by the clinical status of the child. After 48 h, LFO could be given as per local preference or per WHO guideline [2], but use of HFNT was not permitted.

#### Data management and statistical methods

Data were collected on case report forms and entered into a database written in OpenClinica version 3. As previously reported [18], protocol adherence within the first 48 h of randomisation was measured as the number and percentage of patients commencing on the randomised treatment, and the number and percentage changing treatment mode before 48 h, overall and by reason (in patients commencing the randomised treatment). The current secondary analyses are reported using descriptive statistics by stratum and/or study arm but were not formally tested for differences between groups. Respiratory support, respiratory distress over time, and respiratory failure at 48 h from randomisation are reported using counts and percentages or as medians and interquartile ranges (IQR) as appropriate. Resource use is reported using medians and IQRs. Physiology parameters (respiratory rate and heart rate) are summarised over time using graphical methods.

## Results

Detailed baseline characteristics have been reported previously by randomisation arms [18] and are summarised by stratum in Table 1. Compared to the moderate hypoxaemia stratum, children enroled in the severe hypoxaemia stratum were younger, were more likely to be cyanosed, and had higher frequencies of pneumonia on chest X-ray, compensated shock, and altered conscious level. As reported in the main trial publication [18], overall adherence to the randomisation strategy was excellent (Table 2) and 48-h mortalities in the severe hypoxaemia stratum (the primary endpoint) were 18/194 (9.3%) in the HFNT arm and 26/194 (13.4%) in the LFO arm. In the (moderate hypoxaemia stratum, 4/363 (1.1%), 9/363 (2.5%) and 10/724 (1.4%) children in HFNT, LFO, and permissive hypoxaemia arm, respectively, had died by 48 h (see Additional File: Figs. S3a and S4 Kaplan–Meier curves for mortality to day 28). Most deaths in both strata occurred before 48 h. Few had aspiration events (8 in total 3 in HFNT arms, 1 in LFO and 5 in control arm) (Additional File: Table S5). Other reported adverse events are listed in Additional File: Table S6.

**Table 1:** Baseline characteristics of children by study stratum

	**Severe hypoxaemia stratum** **(SpO**_**2**_** < 80%)** ***N* = 388**	**Moderate hypoxaemia stratum** **(SpO**_**2**_** 80 to < 92%)** ***N* = 1454**
Parameter		
Median age (IQR) months	7 (2–19)	9 (4–24)
Male sex *n* %	190 (49)	849 (58.4)
Median SpO_2_ (IQR)	75 (68–78)	88 (85–90)
SpO_2_ < 70% (severe hypoxaemia stratum) or < 85% (hypoxaemia stratum) *n* %	110 (28.3)	300 (20.6)
Median weight (IQR) kg	7 (5–9.5)	8 (6–11)
Median mid upper arm circumference (IQR) cm	13 (11–14)	14 (13–15)
Fever (axillary temperature > 37.5 °C) *n* %	211 (54.3)	763 (52.5)
Hypothermia (re < 36 °C) *n* %	18 (4.6)	33 (2.3)
Severe tachypnoea^a^ *n* %	383 (98.7)	1448 (99.7)
Indrawing *n*/*N* %	373/386 (96.6)	1335 (91.8)
Cyanosis *n*/*N* %	28/386 (7.3)	10 (0.7)
Crepitations *n*/*N* %	285/387 (73.6)	1068/1453 (73.5)
Wheeze *n*/*N* %	79/387 (20.4)	366/1451 (25.2)
Pneumonia signs on chest X-ray *n*/*N* %	241/320 (75.3)	870/1384 (62.9)
Severe tachycardia *n*/*N* %	149 (38.4)	367/1452 (25.3)
Compensated shock *n*/*N* %	239/387 (61.8)	571 (39.3)
Conscious level: responds to		
Pain or voice *n*/*N* %	57 (14.7)	54 (3.7)
Unresponsive *n*/*N* %	21 (5.4)	8 (0.6)
Median haemoglobin (IQR) g/dl	10 (8–11)	10 (9–12)
Severe anaemia (Hb < 5/dl) *n*/*N* %	35 (9)	116 (8)
Median white cell count (10 × 3/_u_L) (IQR)	13 (9–20)	12 (8–17)
Leucocytosis (WBC > 11) *n*/*N* %	228/366 (62.3)	756/1395 (54.2)
Lactate > 5 mmol/L *n*/*N* %	79/381 (20.7)	109/1408 (7.7)
Bacteraemia *n*/*N* %	17/370 (4.6)	34/1409 (2.4)
HIV^b^ *n* %	17/380 (4.5)	32/1422 (2.3)
Malaria RDT^c^ *n*/*N* %	43/373 (11.5)	185/1411 (13.1)
Malaria slide positive *n* %	24/371 (6.5)	77/1413 (5.5)
Severely malnourished *n*/*N* %	39/387 (10.1)	67/1451 (4.6)
Sickle cell disease *n*/*N*^c^ (%)	30/388 (7.7)	154/1454 (10.6)
Known cardiac condition	5 (1.3)	7 (0.5)
Developmental delay *n*/*N* (%)	32/387 (8.3)	78/1452 (5.4)

Defined using FEAST shock criteria: one or more signs of capillary refill > 2 s; lower limb temperature gradient; weak radial pulse volume; severe tachycardia

^a^Defined using FEAST tachypnoea criteria: If aged < 1 year > 34; 1–5 years > 22; and > 5 years > 18 breaths per minute

^b^Defined using FEAST tachycardia criteria If aged < 1 year > 180, 1–5 years > 160 and > 5 years > 140 beats per minute

^c^Known or by genotype (batched genotype at the end of the trial)

**Table 2:** Adherence to protocol

	Severe hypoxaemia stratum COAST A		Moderate hypoxaemia stratum COAST B		
	HFNT	Low-flow	HFNT	Low-flow	Control
HFNT not initiated (died before initiation)	2^a^		1^a^		
Per protocol					
Change of HFNT to low flow < 48 h	1^c^		3^c^		
Control arm low flow (for SpO_2_ < 80%)					104/726
Protocol deviation					
Any use of HFNT in low flow arm		0		2^b^	0
Initiation of low flow oxygen at SpO_2_ ≥ 80%					3

^a^2 patients in COAST A and 1 in COAST B randomised to HFNC died less than 30 min following randomisation and did not commence any respiratory support

^b^2 patients in COAST B randomised to low-flow started on HFNC

^c^including 3 who were not able to tolerate HFNT (defined as severe agitation, pulling off the nasal cannulae) and 1 with nasal trauma)

### Respiratory support and weaning

We generated granular data over the first 48 h on flow rate, FiO_2_, litres of oxygen, and proportion weaned for the respective strata (Tables 3, 4, and 5) from bedside observation of oxygen administration. Deaths over time (indicated in (*italics*)) also resulted in fewer observations in the later timepoints.

**Table 3:** Respiratory support: flows rates, oxygen usage and weaning over 48 h by stratum and study arm. Severe hypoxaemia stratum (saturations < 80% at enrolment)

Hours from start	Low flow						HFNT						
	***N* evaluable** ***(n died)***	*N* (%) in receipt	Median (IQR) flow rate	Median (IQR) FiO_2_	Median (IQR) additional O_2_ l/min	Weaned *N* (%)	***N* evaluable** ***(n died)***	*N* (%) with any support	*N* (%) receiving with room air only	Median (IQR) flow rate	Median (IQR) FiO_2_	Median (IQR) additional O_2_ l/min	Weaned *N* (%)
0.00	194	194 (100)	1 (1, 2)	96 (95, 97)	1.0 (1.0, 2.0)	0 (0.0)	194 *(1)*	194 (100.0)	145 (74.7)	14 (10, 20)	21 (21, 30)	0.0 (0.0, 0.7)	0 (0.0)
0.25	194	194 (100)	1 (1, 2)	96 (95, 97)	1.0 (1.0, 2.0)	0 (0.0)	193 *(1)*	193 (100.0)	143 (74.1)	14 (10, 20)	21 (21, 30)	0.0 (0.0, 0.7)	0 (0.0)
0.50	194	194 (100)	1 (1, 2)	96 (95, 97)	1.0 (1.0, 2.0)	0 (0.0)	192	192 (100.0)	87 (45.3)	14 (10, 20)	30 (21, 30)	0.9 (0.0, 1.6)	0 (0.0)
0.75	194	194 (100)	2 (1, 2)	96 (95, 97)	1.5 (1.0, 2.0)	0 (0.0)	192	192 (100.0)	44 (22.9)	14 (10, 20)	30 (30, 30)	1.3 (0.7, 2.1)	0 (0.0)
1	194 *(2)*	186 (95.9)	2 (1, 2)	96 (95, 97)	1.5 (1.0, 2.0)	8 (4.1)	192 *(2)*	192 (100.0)	55 (28.6)	14 (9, 20)	30 (21, 35)	1.4 (0.0, 2.2)	0 (0.0)
2	192 *(1)*	160 (83.3)	2 (1, 3)	96 (95, 97)	1.5 (1.0, 2.5)	32 (16.7)	190 (*2)*	187 (98.4)	43 (22.6)	14 (9, 20)	30 (25, 30)	1.3 (0.5, 2.1)	3 (1.6)
4	191 *(5)*	161 (84.3)	2 (1, 2)	96 (95, 97)	1.5 (1.0, 2.0)	30 (15.7)	188 *(4)*	174 (92.6)	38 (20.2)	13 (9, 19)	30 (25, 30)	1.3 (0.6, 2.1)	13 (6.9)
8	186 (*2)*	154 (82.8)	2 (1, 3)	96 (95, 97)	1.5 (1.0, 2.5)	32 (17.2)	184 *(2)*	159 (86.4)	43 (23.4)	12 (8, 18)	30 (21, 35)	1.3 (0.0, 2.2)	24 (13.0)
12	184 (*4)*	147 (79.9)	2 (1, 3)	96 (95, 97)	2.0 (1.0, 2.5)	37 (20.1)	182 *(2)*	153 (84.1)	40 (22.0)	12 (8, 18)	30 (21, 30)	1.1 (0.0, 2.1)	28 (15.4)
24	180 *(14)*	134 (74.4)	2 (1, 2)	96 (95, 97)	1.5 (1.0, 2.0)	46 (25.6)	180 *(4)*	150 (83.3)	45 (25.0)	12 (8, 17)	30 (21, 35)	1.1 (0.0, 1.9)	30 (16.7)
48	166	98 (59.0)	2 (1, 3)	96 (95, 97)	1.5 (1.0, 2.5)	68 (41.0)	176	117 (66.5)	41 (23.3)	10 (7, 14)	30 (21, 30)	1.0 (0.0, 1.6)	59 (33.5)

**Table 4:** Respiratory support: flows rates, oxygen usage and weaning over 48 h by stratum and study arm. Moderate hypoxaemia stratum (saturations 80–91% at enrolment) low flow and high flow arms

Hours from start	Low flow						HFNT						
	***N* evaluable** ***(n died)***	*N* (%) receiving	Median (IQR) flow rate	Median (IQR) FiO_2_	Median (IQR) additional O_2_ l/min	Weaned *N* (%)	***N* evaluable *(n died)***	*N* (%) receiving any	*N* (%) receiving with room air only	Median (IQR) flow rate	Median (IQR) FiO_2_	Median (IQR) additional O_2_ l/min	Weaned *N* (%)
0.00	364	364 (100.0)	1 (1, 2)	96 (95, 97)	1.0 (1.0, 2.0)	0 (0.0)	363	363 (100.0)	360 (99.2)	16 (13, 22)	21 (21, 21)	0.0 (0.0, 0.0)	0 (0.0)
0.25	364	363 (99.7)	1 (1, 2)	96 (95, 97)	1.0 (1.0, 2.0)	0 (0.0)	363 (*1)*	363 (100.0)	362 (99.7)	16 (13, 22)	21 (21, 21)	0.0 (0.0, 0.0)	0 (0.0)
0.50	364	362 (99.5)	1 (1, 2)	96 (95, 97)	1.0 (1.0, 2.0)	1 (0.3)	362	361 (99.7)	354 (97.8)	16 (13, 22)	21 (21, 21)	0.0 (0.0, 0.0)	0 (0.0)
0.75	364 *(1)*	361 (99.2)	1 (1, 2)	96 (95, 97)	1.0 (1.0, 2.0)	2 (0.5)	362	361 (99.7)	285 (78.7)	16 (12, 22)	21 (21, 21)	0.0 (0.0, 0.0)	0 (0.0)
1.00	363 *(1)*	286 (78.8)	1 (1, 2)	96 (95, 97)	1.0 (1.0, 2.0)	76 (20.9)	362	355 (98.1)	275 (76.0)	15 (9, 20)	21 (21, 21)	0.0 (0.0, 0.0)	6 (1.7)
2.00	362 *(1)*	213 (58.8)	1 (1, 2)	96 (95, 97)	1.0 (1.0, 2.0)	148 (40.9)	362	299 (82.6)	213 (58.8)	16 (11, 21)	21 (21, 25)	0.0 (0.0, 0.7)	62 (17.1)
4.00	361 *(1)*	178 (49.3)	1 (1, 2)	96 (95, 97)	1.0 (1.0, 2.0)	182 (50.4)	362	232 (64.1)	149 (41.2)	15 (9, 19)	21 (21, 30)	0.0 (0.0, 1.1)	129 (35.6)
8.00	360 *(1)*	160 (44.4)	1 (1, 2)	96 (93, 97)	1.0 (1.0, 2.0)	200 (55.6)	362 (*1)*	197 (54.4)	118 (32.6)	14 (9, 19)	21 (21, 30)	0.0 (0.0, 1.5)	164 (45.3)
12.00	359	141 (39.3)	1 (1, 2)	96 (95, 97)	1.0 (1.0, 2.0)	218 (60.7)	*361 (2)*	177 (49.0)	105 (29.1)	14 (9, 19)	21 (21, 30)	0.0 (0.0, 1.4)	182 (50.4)
24.00	359 *(9)*	129 (35.9)	1 (1, 2)	96 (93, 97)	1.0 (1.0, 2.0)	230 (64.1)	359 *(1)*	161 (44.8)	92 (25.6)	12 (9, 18)	21 (21, 30)	0.0 (0.0, 1.4)	197 (54.9)
48.00	350	92 (26.3)	1 (1, 2)	96 (90, 97)	1.0 (0.5, 2.0)	258 (73.7)	358	115 (32.1)	76 (21.2)	10 (7, 16)	21 (21, 30)	0.0 (0.0, 1.0)	243 (67.9)

**Table 5:** Respiratory support: flows rates, oxygen usage and weaning over 48 h by stratum and study arm. Moderate hypoxaemia stratum (saturations 80–91% at enrolment) permissive hypoxaemia arm

**Hours from start**	*N* evaluable ***(n died)***	*Switched to Low Flow (per protocol)*				*N* not receiving (%)
0.00	727 (1)	0 (0.0)				727 (100.0)
0.25	726	2 (0.3)	1 (1, 1)	93 (87, 98)	1.0 (1.0, 1.0)	724 (99.7)
0.50	726	20 (2.8)	1 (1, 1)	96 (91, 98)	1.0 (1.0, 1.0)	706 (97.2)
0.75	726	30 (4.1)	1 (1, 2)	96 (70, 98)	1.0 (1.0, 1.5)	696 (95.9)
1.00	726	41 (5.6)	1 (1, 2)	96 (87, 98)	1.0 (1.0, 2.0)	685 (94.4)
2.00	726	70 (9.6)	1 (1, 2)	96 (91, 97)	1.0 (1.0, 2.0)	656 (90.4)
4.00	726 (1)	74 (10.2)	1 (1, 2)	96 (90, 97)	1.0 (1.0, 2.0)	652 (89.8)
8.00	725 (1)	75 (10.3)	1 (1, 2)	96 (95, 97)	1.0 (1.0, 2.0)	650 (89.7)
12.00	724 (7)	73 (10.1)	1 (1, 2)	96 (95, 97)	1.0 (1.0, 2.0)	651 (89.9)
24.00	717 (12)	71 (9.9)	2 (1, 2)	96 (95, 97)	1.5 (1.0, 2.0)	646 (90.1)
48.00	705	59 (8.4)	2 (1, 2)	96 (95, 97)	1.5 (1.0, 2.0)	646 (91.6)

#### Severe hypoxaemia stratum

In the low flow arm, all children started at a median flow rate of 1 L/min (IQR 1–2) with an estimated FiO_2_ of 96% (95–97%) increasing to a median of 2 L/min (IQR 1–2) at 2 and 8 h. Thirty-two (17%) had been weaned by 8 h. By 48 h, 68 (41%) had been weaned while 98/166 (59%) remained on oxygen. In the HFNT arm, 145/194 (74.7%) started on room air only (FiO_2_ 21%) at a rate of 14 L/min (IQR 10–20. By 2 h, the proportion receiving respiratory support in room air alone had dropped to 43/181 (23%). In those receiving a blend of air and oxygen, the median flow rate was 14 (IQR 9–20) of which additional oxygen usage was 1.1 L/h (median FiO_2_ 30% (IQR 25–30%)). By 8 h, of those receiving respiratory support, 25% remained in room air and 24 (12.6%) children had been weaned. At 48 h, 128 (68%) children were still receiving respiratory support; 59 (34%) had been weaned.

#### Moderate hypoxaemic stratum

In the LFO arm, 364 children started at a median (IQR) low rate of 1 l/m (1-2) with an estimated FiO_2_ 98% (98–98%). By 2 h, a high number had been weaned 148 (41%). By 8 h, the proportion weaned was 200/360 (55.6%). For those receiving oxygen, median flow rates and FIO_2_ were similar at all timepoints*.* At 48 h, only 92/350 (26%) still required oxygen in the LFO arm. In the HFNT arm, 360/363 (99.2%) started this in room air (with no additional oxygen) with a median (IQR) flow rate of 16 (13-22) but by 1 h, 24% had added oxygen. By 2 h, when weaning was permitted per protocol, 62 (17%) were weaned and 213/362 (59%) remained on respiratory support with HFNT in room air alone; 86 had HFNT with blended oxygen (median flow rate 16 (13-32) at 1 l/h) and one child had switched to LFO. By 8 h, 164 (45%) had been weaned, and 195/358 (54.5) remained on respiratory support. Of these, 117 (60%) were on HFNT in room air alone, 78 on HFNT with blended oxygen, median (IQR) FiO_2_ 30% (21–30%). At 48 h, 116/360 (32%) children remained on high flow who were then switched to low flow (per protocol). In the permissive hypoxaemia (control) arm, all children started on no supplemental oxygen; by 1 h and 2 h respectively, 41 (5.6%) and 70 (9.6%) had started on LFO due to desaturations to less than 80%. By 24 and 48 h, the overall proportion requiring oxygen remained at 10% (saturations < 92%) and at 48 h, these children were switched to LFO.

### Respiratory failure at 48 h

A secondary endpoint of the main trial was respiratory failure (defined as the presence of hypoxaemia (an oxygen saturation of < 92% and increased work of breathing (respiratory distress) at 48 h)). In the severe hypoxaemia stratum, respiratory failure was present in 15/175 (8.6%) in the HFNT arm vs 18/167 (10.8%) in the LFO arm. In the moderate hypoxaemia stratum, the respective numbers with respiratory failure in the combined liberal oxygenation strategies were 5/359 (1.4%) vs 8/353 (2.3%) in the control. These and associated co-morbidities by stratum are presented in Table 6. In the severe hypoxaemia stratum, 17/33 (51.0%) had radiological signs (or a clinically history) of congenital heart disease (or cardiac failure). Cardiac abnormalities were also prevalent in the moderate hypoxaemia stratum and, to a lesser extent, comorbidities with sickle cell disease, HIV or pulmonary tuberculosis.

**Table 6:** Respiratory failure at 48 h by stratum

	Severe hypoxaemia	Moderate hypoxaemia	Total
Alive at 48 h	342	1423	1765
Respiratory failure	33 (9.6%)	46 (3.2%)	79
Proportion remaining ≤ 80%	14 (42%)	4 (8.7%)	18
Co-morbidities			
Cardiac^a^	17 (51.5%)	9 (19.5%)	26
HIV/TB (either or both)	7 (21.2%)	3 (6.5%)	10
Sickle cell disease	0	3 (6.5%)	3

^**a**^Congenital heart disease (*n*−16) or cardiac failure (*n* = 2)

### Bedside vital signs over time

**Fig. 1:**
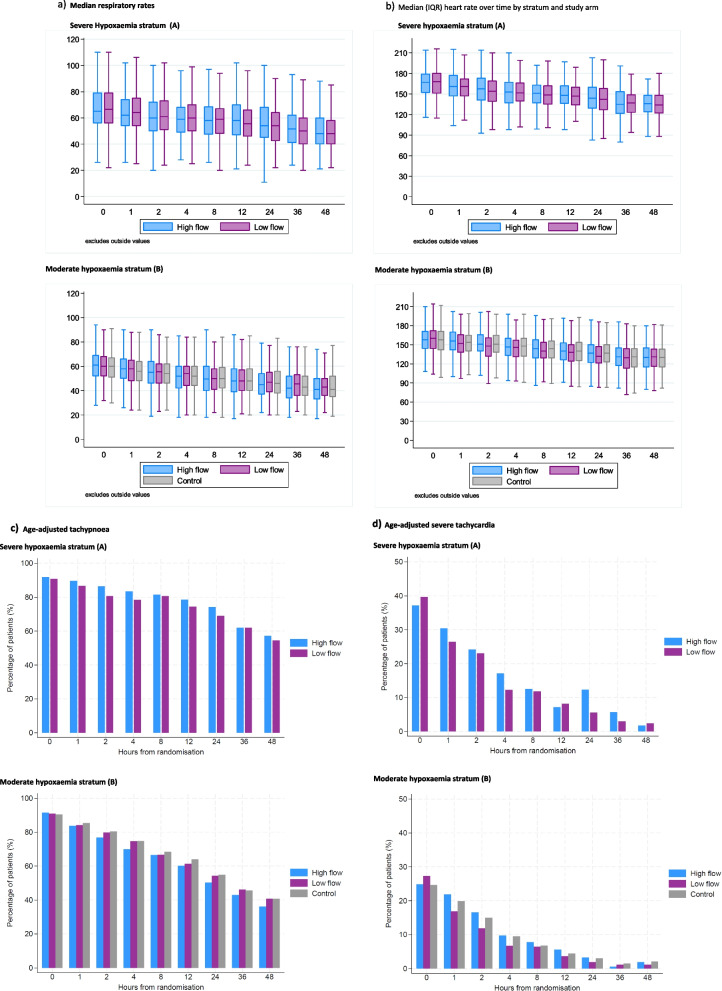
Changes in bedside vital signs over time by stratum and arm. **a** Median respiratory rates: (A) severe hypoxaemia stratum, (B) moderate hypoxaemia stratum. **b** Median (IQR) heart rate over time by stratum and study arm: (A) severe hypoxaemia stratum, (B) moderate hypoxaemia stratum. **c** Age-adjusted tachypnoea: (A) severe hypoxaemia stratum, (B) moderate hypoxaemia stratum. **d** Age-adjusted severe tachycardia: (A) severe hypoxaemia stratum, (B) moderate hypoxaemia stratum

Within each stratum, by arm, we compared changes in median respiratory and heart rates over time and age-adjusted proportion of children with tachypnoea and tachycardia (Fig. 1a–d) and work of breathing over time (Table 7), respectively. On enrolment (baseline) most children had severe tachypnoea and, as expected, all had increased work of breathing (defined as indrawing or deep breathing). The median respiratory and heart rates over time were similar across all arms by stratum, including in the permissive hypoxaemia arm, within COAST B stratum. Over time, there were improvements in the proportion without increased work of breathing (recorded by trial clinicians as presence of indrawing), which was similar across each of the strata randomisations. At 48 h in the respective strata, 77–80% and 53–63% still had respiratory distress but without hypoxaemia and were thus not receiving oxygen.

**Table 7:** Presence of respiratory distress over time

Respiratory distress	1	2	4	8	12	24	36	48
*HFNT Arm*								
Evaluable	192	190	188	184	182	180	177	176
% present	98.96	97.89	95.21	93.48	91.21	88.33	83.62	77.27
*Low Flow Arm*								
Evaluable	194	192	191	186	184	180	170	166
% present	97.42	97.92	95.81	97.31	94.57	90.56	88.82	80.72
Moderate hypoxaemia stratum (saturations <80–91% at enrolment)								
*HFNT Arm*								
Evaluable	362	362	362	362	361	359	358	358
% present	96.13	93.92	90.33	87.29	82.27	74.37	65.64	53.63
*Low flow arm*								
Evaluable	363	362	361	360	359	359	355	350
% present	96.97	96.69	93.63	89.17	84.96	77.72	68.45	63.14
*Permissive hypoxaemia*								
Evaluable	726	726	726	725	724	717	716	705
% present	95.04	93.66	89.12	85.10	82.32	74.48	68.58	58.16

### Resource use by study arm

The mean duration of respiratory support in the severe hypoxaemia strata was 28–30 h, which was considerably longer than that in children on the liberal oxygenation strategies in the moderately hypoxaemia stratum (16 h). However, there were wide ranges around these median values (Table 8). Compared to HFNT, we observed a higher median oxygen use (measured in litres given over 48 h) in the LFO arms: 2743 L (interquartile range 895–4884) versus 19,823 L (IQR 502–3571) for HFN and 480 L (236.2, 2132.2) versus 113 L (0.0–1454) for the respective strata. The permissive hypoxia, in the moderate hypoxaemic stratum, had the lowest use of oxygen therapy, median 0 L (IQR 0–0), with a mean of 306L (s.d. 1080).

**Table 8:** Summary of resource use by study arm and stratum

**Stratum**	Severe hypoxaemia		Moderate hypoxaemia		
**Study arm**	High-flow	Low-flow	High-flow	Low-flow	Permissive hypoxaemia
Hospital Length of stay (days), median (IQR)	5 (3, 8)	5 (3, 7)	4 (3, 5)	4 (3, 6)	4 (3, 5)
Hours of respiratory support in first 48 h, median (IQR)	36.6 (8.8, 48)	32.1 (7.4, 47.7)	8.4 (2.7, 26.8)	6.8 (2.5, 25.3)	0 (0,0)
Hours receiving additional oxygen in first 48 h, median (IQR)	28.6 (5.8, 46.6)	32.1 (7.4, 47.7)	1.0 (0.0, 18.7)	6.8 (2.5, 25.3)	0.0 (0.0, 0.0)
Litres of oxygen used in first 48 h, median (IQR)	1982.9 (502.2, 3571.2)	2742.5 (894.5, 4883.5)	113.4 (0.0, 1453.9)	480.0 (236.2, 2132.2)	0.0 (0.0, 0.0)
Oxygen l/min in first 48 h, median (IQR)	1.0 (0.0, 1.8)	1.5 (1.0, 2.0)	0.0 (0.0, 0.0)	1.0 (1.0, 2.0)	1.0 (1.0, 2.0)

## Discussion

Approximately 80% of children presenting with severe pneumonia who were enroled into the COAST trial had moderate hypoxaemia (SpO_2_ 80–92%). Within this stratum, only 50% of children randomised to liberal strategies required oxygen and/or respiratory support for a maximum of 8 h. Even though signs of respiratory distress persisted to 48 h, this was not associated with hypoxaemia and thus children remained off oxygen therapy. The ‘oxygen-sparing’ HFNT protocol implemented within this trial, providing respiratory support with a minimal level of titrated blended oxygen, resulted in substantially lower volumes of oxygen consumption over 48 h compared to the amount received in the low flow strategy. Thus, over this period even in the most liberal of the strategies (LFO) the median volume of oxygen in the respective strata (2743 L and 480 L/child) was, as a result of the trial protocol, substantially lower than in a LFO equivalent strategy (5990 L over a median of 2.5 days) reported in the multicentre study in Nigeria investigating oxygen use in children with pneumonia [19]. In the permissive arm, minimal volumes of oxygen were used overall. Only 10% required oxygen at any time, but these were largely identified within 2 h of enrolment. In children classified as having respiratory failure (persistent oxygen requirement) at 48 h, over 70% had either an underlying cardiac co-morbidity (largely uncorrected congenital heart disorder or decompensated heart failure) [20] or the complications of HIV or tuberculosis, indicating that those with persistent or refractory hypoxaemia should be investigated for additional co-morbidities.

Major gaps exist between current treatment policies and the implementation of the potentially life-saving role of oxygen in clinical management, largely stemming from the lack of relevant research. Consequently, current treatment recommendations are based on weak evidence. The COAST trial was designed to address the evidence gaps in oxygen therapy in children with severe pneumonia. The design also reflected the contextual realities of the delivery of oxygen and respiratory support that have been widely reported across Africa. On the demand side, since children hospitalised with severe pneumonia remain a major public health concern, they constitute a substantial burden to the under-resourced health services for the adequate provision of oxygen [21]. On the supply side, despite over two decades of investment by WHO technologies groups aimed at improving systems for delivering oxygen, this component of care appears not to have been afforded a high enough priority at both global and national levels. The issue of ‘oxygen insecurity’ remains a major barrier to the widespread implementation of current recommendations for severe pneumonia in hospitals with limited resources [21]. Local data from East Africa corroborated these key barriers to oxygen implementation. In a 2019, a Ugandan survey of private and non-public hospitals (using WHO Tool for Situational Analysis to Assess Emergency and Essential Surgical Care) found that only 37.5% of hospitals had oxygen for over 25% of the time [22]. This fact was echoed in an assessment of healthcare-provider perceptions of barriers to oxygen therapy [23].

One of the limitations of this study is that at present we have a poor understanding of the pathogenic aetiology of the pneumonias despite 75% and 63% having radiographic evidence of pneumonia. Few had documented bacteraemia; however, many (between 56 and 63%) were receiving antibiotics prior to or at admission [24]. Nevertheless, the data remain pertinent to the undifferentiated child with severe pneumonia in East Africa owing to the multi-centre nature of the trial, including both children living in urban and rural areas. An additional limitation is that whilst we have presented granular details on the oxygen use across the three strategies, we have presented no cost implications for health services. This will be covered in a separate manuscript, including a detailed cost-consequence analysis.

Oxygen therapy in African hospitals is probably one of the most poorly ‘regulated’ drugs. It is rarely formally prescribed in the treatment chart, infrequently monitored for response, and early weaning is challenged by the variable availability of pulse oximetry. Our trial demonstrated children could be successfully weaned from oxygen once hypoxaemia has corrected. For example, in 50% of children with moderate hypoxaemia (the majority (79%) of children with severe pneumonia in this study) normalisation of oxygen levels had occurred by 8 h. This indicates that the WHO manuals on oxygen therapy recommendations are likely to expose children to hyperoxia and potentially to the harms of oxygen toxicity. Where pulse oximetry is not available, this is almost certainly true. Moreover, the 48 h and day 28 mortality in children enroled in the respective strata in the COAST trial (including children in the permissive hypoxaemia arm) were substantially lower than had been reported from the continent, including children in the permissive hypoxaemia arm and from our pre-trial data informing the relevant trial strata [19, 25, 26].

Finally, the standard methods of humidification for medical oxygen in many hospitals in Africa are inadequate to minimise the risks of administration of medical oxygen. The current oxygen treatment manual indicates *that humidification is not required* for flow rates of 1–4 l/min when administered via nasal prongs or cannula (strong recommendation (low quality of evidence) [2]. Where humidification is used, it is generally at room temperature (without warming) and thus inadequate to reliably humidify oxygen. Compressed medical oxygen stored in standard cylinders represents one of the driest gases on earth. WHO guidance on medical oxygen [27, 28] states that medical oxygen may contain a maximum water content of 67 parts per million, which translates to a dew point of − 45.5 °C, meaning it has very close to 0% relative humidity. By the time it reaches the child’s lower airway, the gas will have a dew point (roughly translating to humidity level) of 37 °C, and the thermal cost of conditioning the gas will have been ‘paid’ by the child. The higher the flow rate, the higher ‘the cost paid’ by the child. At a flow of 5 l/min, unconditioned medical oxygen could absorb, from the patient’s airway (a very small part of their anatomy), up to a litre of water every 3 days. We can find no data comparing the current standard of practice (recommended by WHO) and oxygen provision alongside adequate humidification.

Whilst oxygen has been used in the treatment of pneumonia for a large part of the last century, the recognition of pulmonary oxygen toxicity as a problem has been relatively recent [15]. Toxicity is related to the concentration of oxygen and length of exposure [29, 30]. For these reasons, other than emergency usage of higher levels of FiO_2_, it is recommended that inspired oxygen concentration (FiO_2_) should be carefully titrated against SpO_2_.

## Conclusion

These data on the rationale use of oxygen therapy and knowledge acquired from the COAST trial should stimulate further debate around the safe provision of oxygen therapy in resource-poor settings. Providing oxygen without a reliable means to monitor it should not be the default. Pulse oximetry should be mandated as a requirement for prescribing medical oxygen for periods longer than a couple of hours.

## Supplementary Information

Supplementary Material 1: Figure S1 Relationship between baseline oxygen saturation and 48-hour mortality in 36,036 Kilifi paediatric general admissions. Figure S2 Relationship between baseline oxygen saturation and 48-hour mortality in the FEAST control arm (*n*=1007). Table S1 Day 2 mortality rates (Kilifi admission data) in children with COAST inclusion criteria. Table S2 Weight-banded Flow rates recommended for Optiflow. Table S3a-c: Protocol for High Flow Nasal Therapy using AirVO (HFNT) therapy and two stepdown titration. Table S4: Low flow Initiation and weaning. Table S5: Numbers with Aspiration Events. Figure S3: Stratum A Kaplan Meier Curve mortality to Day 28 by Arm. Figure S4: Stratum B Kaplan Meier Curve mortality to Day 28 by Arm. Table S6 Line list of adverse events/other complications.

## Data Availability

KEMRI Wellcome Trust Programme supports a controlled access approach based on completion of a data request proforma available from the lead author (k.maitland@imperial.ac.uk).

## Abbreviations

COAST: Children’s Oxygen Administration Strategies Trial
HFNT: High flow nasal therapy
IQR: Interquartile range
KWTRP: KEMRI Wellcome Trust Research Programme
LFO: Low flow oxygen
RDT: Rapid diagnostic test
SD: Standard deviation
SpO_2_: Percentage of oxygen in the blood measured by a pulse oximeter
WHO: World Health Organization
