# Supplementary material for: Early weaning from oxygen therapy in African children with severe pneumonia
Source: BMC Med. 2025 Jul 1;23:366. doi: 10.1186/s12916-025-04178-9 (PMC12220327; doi:10.1186/s12916-025-04178-9)
Supplement: Supplementary file 1 — Supplementary Material 1: Figure S1 Relationship between baseline oxygen saturation and 48-hour mortality in 36,036 Kilifi paediatric general admissions. Figure S2 Relationship between baseline oxygen saturation and 48-hour mortality in the FEAST control arm (n=1007). Table S1 Day 2 mortality rates (Kilifi admission data) in children with COAST inclusion criteria. Table S2 Weight-banded Flow rates recommended for Optiflow. Table S3a-c: Protocol for High Flow Nasal Therapy using AirVO (HFNT) therapy and two stepdown titration. Table S4: Low flow Initiation and weaning. Table S5: Numbers with Aspiration Events. Figure S3: Stratum A Kaplan Meier Curve mortality to Day 28 by Arm. Figure S4: Stratum B Kaplan Meier Curve mortality to Day 28 by Arm. Table S6 Line list of adverse events/other complications. [file 12916_2025_4178_MOESM1_ESM.pdf]

Supplement

Background

Risk of mortality modelled against oxygen saturation

We examined the relationship between oxygen saturation at admission (or baseline) and mortality by 48 hours (or discharge date), using a fractional polynomial logistic model restricted to the baseline measure, in the Kilifi (KEMRI Wellcome Trust Programme) dataset of paediatric general admissions (n=36,036; unselected admissions) (**Figure S1**). The same relationship was also modelled and examined in more critically ill children using the control arm group of the FEAST Trial[1] (n=1007) (**Figure S2**). The trial eligibility criteria included children with severe illness (and shock), thus is equivalent to the inclusive definition of VSP (respiratory distress (83%) and/or impaired consciousness (72%). Both datasets indicated a steady increase of risk of mortality as SpO_2 decreases from 100% to 80%, with an inflection of the risk of mortality at around 80%. The wider 95% CIs at low values in the FEAST dataset are due to low numbers but is less pronounced for SpO_2 ≥80% (unpublished data). Relevant to the design of the COAST trial is within the range 80-92% where the oxygen-attributable preventable mortality (assuming that oxygen does prevent mortality) is very low because the mortality across this range of saturations is relatively flat. Within this subgroup, the mortality remains relatively constant across the upper to lower thresholds (1-2%) difference with no significant difference between the higher and lower threshold seen (see **Table S1**Therefore, any risk from giving or initially withholding oxygen to the population of children targeted in the COAST trial will be low.

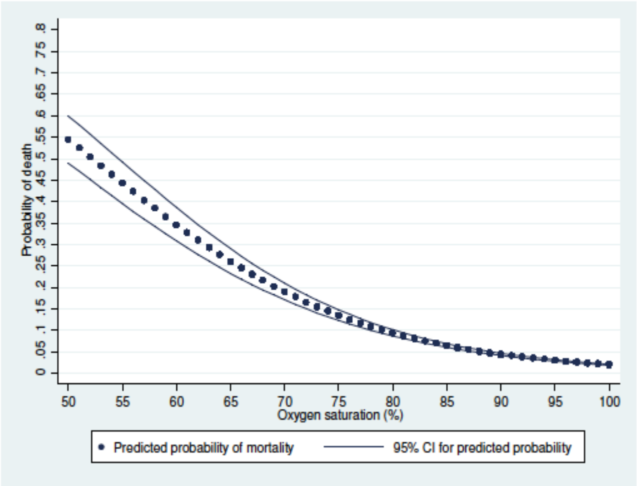

Figure S1 Relationship between baseline oxygen saturation and 48-hour mortality in 36,036 Kilifi paediatric general admissions

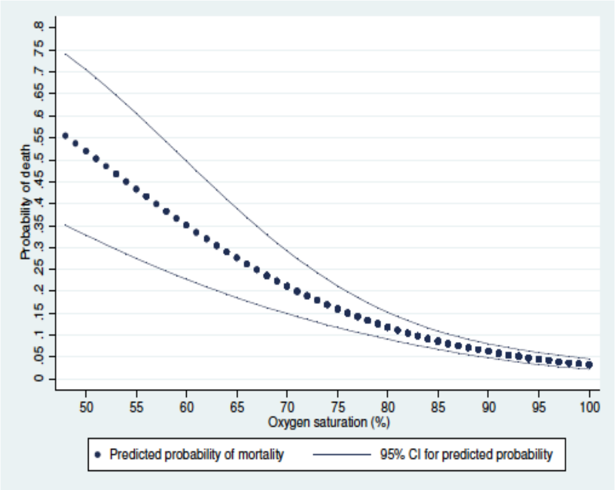

Figure S2 Relationship between baseline oxygen saturation and 48-hour mortality in the FEAST control arm (n=1007)

Table S1 Day 2 mortality rates (Kilifi admission data) in children with COAST inclusion criteria:

**Respiratory presentation* at admission**	**Mortality at 2 days**
Oxygen Saturations	
80-82%	32/240 (13%)
83-86%	26/259 (10%)
87-89%	48/392 (12%)
Total	106/891 (12%)
χ^2 test	P=0.50

*Indrawing or deep breathing

[1]Maitland K, Kiguli S, Opoka RO, Engoru C, Olupot-Olupot P, Akech SO, et al. Mortality after fluid bolus in African childre

Methods

Oxygen administration protocols for COAST

Table S2 Weight-banded Flow rates recommended for Optiflow

Table S3a-c: Protocol for High Flow Nasal Therapy using AirVO (HFNT) therapy and two step down titration

Table S4: Low flow Initiation and weaning

Table S2 Weight-banded Flow rates recommended for Optiflow

Weight	HFNC Flow rates	Comments	FPH Circuit to use
0-12 kg	2L/kg/min	Max 25 L/min	Paediatric circuit
13-15 kg	30L/min		Adult circuit
16-30 kg	35L/min		Adult circuit
31-50 kg	40L/min		Adult circuit
>50 kg	50L/min		Adult circuit

Table S3: Protocol for High Flow AirVO (HFA) therapy

Table S3a Patient interface and Optiflow setting

The appropriate patient interface, breathing circuit and OptiFlow settings were selected based on the manufactures' guidance.

**Weight**	**Circuit Type**	**Patient Interface**	**Optiflow setting**
0 – 12 kgs	Paediatric	Infant (3-12kgs) or pediatric (8- 12kgs) if fit is better	Junior mode
> 12kgs	Adult	Small adult nasal cannula	Adult mode

https://www.fphcare.com/en-gb/products/optiflow-junior-breathing-circuit/
https://www.fphcare.com/en-gb/products/infant-nasal-cannula/

Table S3b Flow rates

Weight	Flow Rate	Maximum flow rate	Mode
**< 12 Kg**	**2 Litres/kg/min**	**25L/min**	**Junior**
**13-15 kg**	**2 L/kg/min**	**30L/min**	**Adult**
**16-30 kg**	**35 L/min**	**40L/min**	**Adult**
**31-50 kg**	**40 L/min**	**50L/min**	**Adult**

Table 3c Protocolised HFNT flow rates and titration recommendations for each clinical assessment

The first 15 mins of HFNT was inititated in room air (21%); every 15mins for the first hour additional oxygen up to a maximum of FiO_2 40% was titrated in to achieve SpO_2≥92% (see tables below).

Time since start of treatment	O2 SATS	Current oxygen/flow setting	Action recommended
**15 MINS**	<80%	HFA (FiO2 21%)	Add in Oxygen (Target FiO2 30%)
	80-91%	HFA (FiO2 21%)	Leave on HFA
	≥92%	HFA (FiO2 21%)	Leave on HFA
**30 MINS**	<80%	HFA (FiO2 21%)<br>HFA (FiO2 30%)	Add in Oxygen (Target FiO2 30%)<br>Add in Oxygen (Target FiO2 35%)
	80-91%	HFA (FiO2 21%)<br>HFA (FiO2 30%)<br>HFA (FiO2 35%)	Leave on current treatment.
	≥92%	HFA (FiO2 21%)<br>HFA (FiO2 30%)<br>HFA (FiO2 35%)	Leave on current treatment
**45 MINS**	<80%	HFA (FiO2 21%)<br>HFA (FiO2 30%)<br>HFA (FiO2 35%)	Add in Oxygen (Target FiO2 30%)<br>Add in Oxygen (Target FiO2 35%)<br>Leave on current treatment.
	80-91%	HFA (FiO2 21%)<br>HFA (FiO2 30%)<br>HFA (FiO2 35%)	Leave on current treatment.
	≥92%	HFA (FiO2 21%)<br>HFA (FiO2 30%)<br>HFA (FiO2 35%)	Leave on current treatment.
**1 HOUR**	<80%	HFA + FiO2 30%	Increase Oxygen Target FiO2 35%
		HFA (FiO2 21%)	Add in oxygen Target FiO2 30%
	80-91%	HFA + FiO2 30%	Leave on current treatment
		HFA (FiO2 21%)	Add in Oxygen Target FiO2 30%
	≥92%	HFA + FiO2 30%	Leave on current setting consider 'Down-Titration' protocol at 2hrs
		HFA (FiO2 21%)	Leave on current setting consider 'Weaning' protocol at 2 hrs

Continued

Time since start	O2 SATS	Current oxygen/flow setting	Action recommended
**2 HOURS**	<80%	HFA + FiO2 35%	Increase Oxygen Target FiO2 40%
		HFA + FiO2 30%	Increase Oxygen Target FiO2 35%
		HFA (FiO2 21%)	Add in Oxygen Target FiO2 30%
	80-91%	HFA + FiO2 35%	Leave on current treatment
		HFA + FiO2 30%	Increase Oxygen Target FiO2 35%
		HFA (FiO2 21%)	Add in Oxygen Target FiO2 30%
	≥92%	HFA + FiO2 35%	Refer to 'Down-Titration' protocol
		HFA + FiO2 30%	Refer to 'Down-Titration' protocol
		HFA (FiO2 21%)	Refer to 'Weaning' protocol if also >
**4 HOURS and beyond**	<80%	On High Flow Air (HFA) +	Continue treatment
		On HFA + FiO2 35%	Increase FiO2 to 40%
		On HFA + FiO2 30%	Increase FiO2 to 35%
		On HFA + FiO2 21%	Increase FiO2 to 30%
		On no HFA treatment	Refer to 'Initiation' protocol
	80-91%	On High Flow Air (HFA) +	Continue treatment
		On HFA + FiO2 35%	Increase FiO2 to 40%
		On HFA + FiO2 30%	Increase FiO2 to 35%
		On HFA + FiO2 21%	Increase FiO2 to 30%
		On no HFA treatment	Refer to 'Initiation' protocol
	≥92%	On High Flow Air (HFA) +	Refer to 'Down-Titration' protocol
		On HFA + FiO2 35%	Refer to 'Down-Titration' protocol
		On HFA + FiO2 30%	Refer to 'Down-Titration' protocol
		On HFA + FiO2 21%	Refer to 'Weaning' protocol
		On no HFA treatment	Monitor at next time point

Two step Down- titration protocol

Time from Start of Down Titration	O2 SATS	Action recommended
**0 MINS**	≥92%	Reduce FiO2 by 10%<br>1/ If at 30% then reduce to room air (21%)<br>2/ If at 35% reduce to 25%<br>3/ If at 40% reduce to 30%
**15 MINS**	<80%	Return to the previous FiO2 and consider 'Down-titration' when stabilised
	80-91%	Leave on the current FiO2 setting
	≥92%	Turn off additional oxygen<br>(if already FiO2 21% then go to 'Weaning High Flow')

Time	O2 SATS	CURRENT TREATMENT	ACTION
**30 MINS**	<80%	HFA + additional oxygen	1/ If Sats have worsened since 15 mins then go back to 'General Titration' protocol using increments recommended<br>2/ If the child is on FIO2 35% (trial of [illegible]

		HFA (FiO2 21%)	Target FiO2 30%
	80-91%	HFA + FiO2 30%	Leave on the current FIO2 setting
		HFA (FiO2 21%)	Add in Oxygen Target FiO2 30%
	≥92%	HFA + FiO2 30%	Consider 'Down-Titration' protocol again
		HFA (FiO2 21%)	Consider 'Weaning' protocol

Children eligible for weaning (minimum after 2 hours of therapy) were titrated down initially from oxygen to air alone then weaned of HFNT (flow rate initially halved then stopped). Children remained off oxygen/HFNT if SpO_2 ≥92% (confirmed by BITMOS sat 801+ oximeters) but weaning would be discontinued or restarted again if SpO_2 fell to <92% before 48hours. At 48 hours children still on HFNT requiring supplemental oxygen were switched onto LFO.

If children were not able to tolerate HFNT or developed a complication, the reason was recorded and per-protocol the child was switched to receive low flow oxygen therapy by mask/nasal cannula.

Table S4 Initiation and weaning of low flow oxygen therapy

The method delivery depended on local preference but generally included a short, nasal prong, catheter or mask. Infants started on a flow rate of 1 l/min and children >1 year commenced on 2 l/min O2. The flow rates were titrated up over the first 30min-1 hour against oxygen saturation (to achieve SpO_2 ≥92%) to a maximum of 2l/min in infants and 4 L/min in children if using nasal cannula (see tables below). If higher rates were required children/infants were switched to mask with a reservoir (partial rebreather mask) and oxygen gradually increased to up 15 L/min and titrating to response of saturation measurements. If no response then the flow rate was reduced to minimize exposure.

TIME	ACTION
**0 MINS**	Start on 1 l/min O2 (infants)<br>Start on 2 l/min O2 (children >1 year)

TIME	O2 SATS	ACTION
**15 MINS**	<80%	Add 0.5l/min
	80-91%	Leave on 1 l/min (infants)<br>Leave on 2 l/min (child >1 year)
	≥92%	Leave on 1 l/min (infants)<br>Leave on 2 l/min (child >1 year)
**30 MINS**	<80%	Add 0.5l/min
	80-91%	Leave on 1 l/min (infants)<br>Leave on 2 l/min (child >1 year)
	≥92%	Leave on 1 l/min (infants)<br>Leave on 2 l/min (child >1 year)
**45 MINS**	<80%	Add 0.5l/min
	80-91%	Leave on 1 l/min (infants)<br>Leave on 2 l/min (child >1 year)

	≥92%	Leave on 1 l/min (infants)<br>Leave on 2 l/min (child >1 year)

TIME	O2 SATS	CURRENT TREATMENT	ACTION
**1 HOUR**	<80%	1 l/min	Increase to 1.5 l/min
		1.5 l/min	Increase to 2 l/min
		2 l/min	Increase to 2.5 l/min
		2.5 l/min	Increase to 3 l/min
	80-91%	1 l/min	Increase to 1.5 l/min
		1.5 l/min	Increase to 2 l/min
		2 l/min	Increase to 2.5 l/min
		2.5 /min	Increase to 3 l/min
	≥92%	1 l/min	Leave on current treatment
		1.5 l/min	Leave on current treatment
		2 l/min	Leave on current treatment
		2.5 l/min	Leave on current treatment

**2 HOURS**		3 l/min	Increase to 3.5 l/min if tolerated or switch to oxygen by mask
	80-91%	1 l/min	Increase to 1.5 l/min
		1.5 l/min	Increase to 2 l/min
		2 l/min	Increase to 2.5 l/min
		2.5 l/min	Increase to 3 l/min
		3 l/min	Increase to 3.5 l/min if tolerated or switch to oxygen by mask
	≥92%	1 l/min	Refer to 'Weaning' protocol
		1.5 l/min	Refer to 'Weaning' protocol
		2 l/min	Refer to 'Weaning' protocol
		2.5 l/min	Refer to 'Weaning' protocol
		3 l/min	Refer to 'Weaning' protocol

TIME	O2 SATS	CURRENT TREATMENT	ACTION
**4 HOURS and beyond**	<80%	1l/min	Increase to 1.5 l/min
		1.5 l/min	Increase to 2 l/min
		2 l/min	Increase to 2.5 l/min
		2.5 l/min	Increase to 3 l/min
		3l/min	Increase to 3.5 l/min if tolerated or switch to oxygen by mask
	80-91%	1l/min	Increase to 1.5 l/min
		1.5 l/min	Increase to 2l/min
		2l/min	Increase to 2.5 l/min
		2.5 l/min	Increase to 3 l/min

		3 l/min	Increase to 3.5 l/min if tolerated or switch to oxygen by mask
	≥92%	1l/min	Refer to 'Weaning' protocol
		1.5 l/min	Refer to 'Weaning' protocol
		2l/min	Refer to 'Weaning' protocol
		2.5 l/min	Refer to 'Weaning' protocol
		3 l/min	Refer to 'Weaning' protocol

MAXIMUM FLOW: 4 L/min was the maximum flow rate you can increase to using nasal cannula for children above 1 year. For infants (< 1 year 2l/min is the maximum you can give by nasal cannula). Check for abdominal distension regularly.

Weaning

Children were eligible for weaning after a minimum of 2 hours on oxygen therapy. Weaning from oxygen occurred over 30 mins if SpO_2 ≥92% (confirmed by BITMOS sat 801+ oximeters). Initially the flow rate was halved and saturations rechecked after 15 minutes. If at 30 mins saturations were within acceptable range as show below, then oxygen was discontinued

Children were continued to be monitored and restart LF if SpO_2 fell to <92% before 48hours. Crossover to HFNT was not permitted per protocol.

TIME	O2 SATS	ACTION
**15 MINS**	<80%	Return to previous low flow therapy treatment- re-attempt at next time point
	80-91%	Leave on current treatment
	≥92%	Switch off oxygen

TIME	O2 SATS	CURRENT TREATMENT	ACTION
**30 MINS**	<80%	Oxygen	Refer to 'General Titration' protocol
		Nil oxygen	Refer to 'Initiation' protocol
	80-91%	Oxygen	Refer to 'General Titration' protocol
		Nil oxygen	Refer to 'Initiation' protocol
	≥92%	Oxygen	switch off oxygen and check sats after 15 mins
		Nil oxygen	Monitor at next time point

Results
Table S5: Numbers with Aspiration Events
Figure S3: Stratum A Kaplan Meier Curve mortality to Day 28 by Arm
Figure S4: Stratum B Kaplan Meier Curve mortality to Day 28 by Arm

Table S5: Numbers with Aspiration Events
Table S6 Line list of adverse events/other complications

	COAST A: High-flow	COAST A: Low-flow	COAST B: High-flow	COAST B: Low-flow	COAST B: Permissive hypoxia
Aspiration	2	0	1	1	5

Table S6 Line list of adverse events/other complications

event	COAST A: High-flow	COAST A: Low-flow	COAST B: High-flow	COAST B: Low-flow	
Acute glonerulonephritis	0	1	0	0	0
Anaemia	0	1	2	2	0
Arthritis	0	0	0	1	0
Asthma	0	0	0	0	3
Bone fracture	0	0	1	0	0
Bronchiolitis	1	0	0	2	0
Bronchospasm	0	0	0	0	1
Cerebral Palsy	0	0	0	0	1
Chest infection	0	1	1	1	1
Chronic fever undiagnosed	1	0	0	0	0
Congenital heart disease	0	0	1	0	0
Congestive heart failure	1	1	2	2	0
Dermatitis	0	0	0	0	1
Diarrhoea	0	0	0	0	2
Folliculitis	0	0	0	0	1
Gastroenteritis	1	0	0	0	2
Hemiparesis	0	0	1	0	0
Malaria	0	0	3	2	4
Malnutrition	0	2	1	0	1
Measles	1	0	0	0	1
Oral Candida	0	0	0	1	0
Osteomyelitis	0	0	1	0	0
Other upper respiratory symptom	0	0	0	1	1
Pneumonia	0	1	2	2	10
Pulmonary Hypertension	0	0	0	1	0
Pulmonary Oedema	1	0	0	0	0
Respiratory tract infection	2	0	1	0	5
Septicaemia	2	1	2	0	4
Shortness of breath	0	0	1	1	0

Sickle Cell Anaemia complication	0	0	2	3	1
Skin Abscess	0	0	0	0	1
Stroke	0	0	1	1	0
Thrombocytopenia	0	0	0	1	0
Tuberculosis	0	0	2	0	1

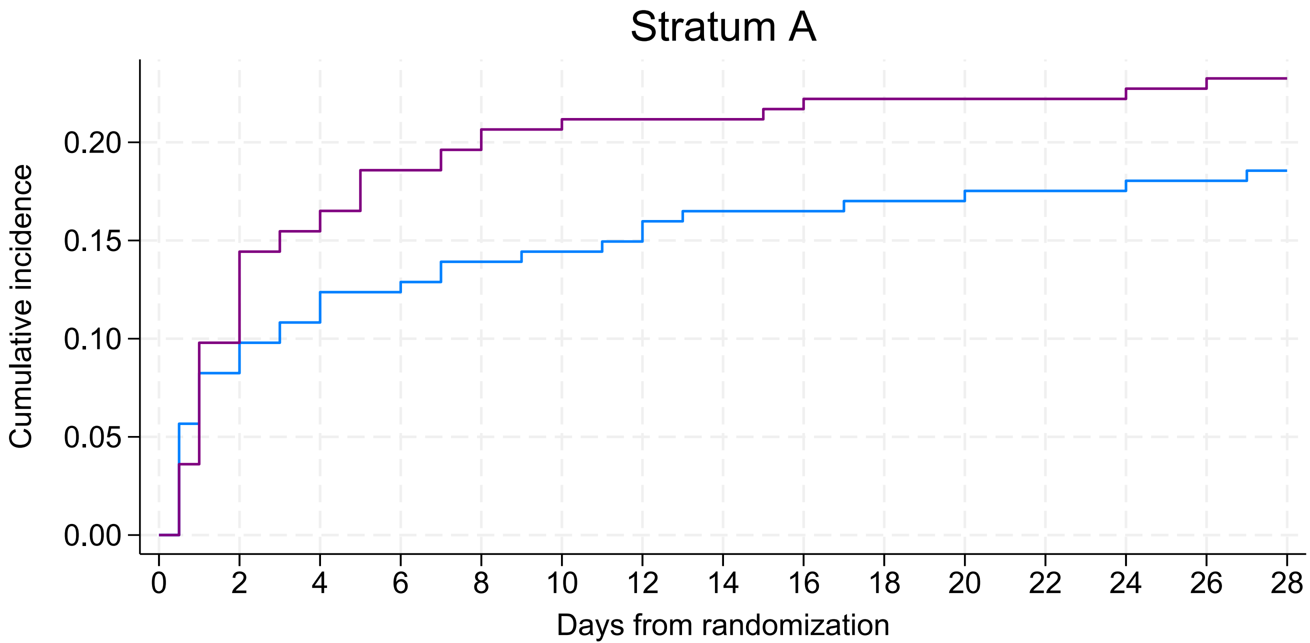

Number at risk

High flow	194	178	173	170	167	166	165	162	162	161	161	160	160	159	158
Low flow	194	175	163	157	155	153	152	152	151	149	149	149	149	148	147

High flow — Low flow

Figure S3 Kaplan Meier Curves for Death to Day 28

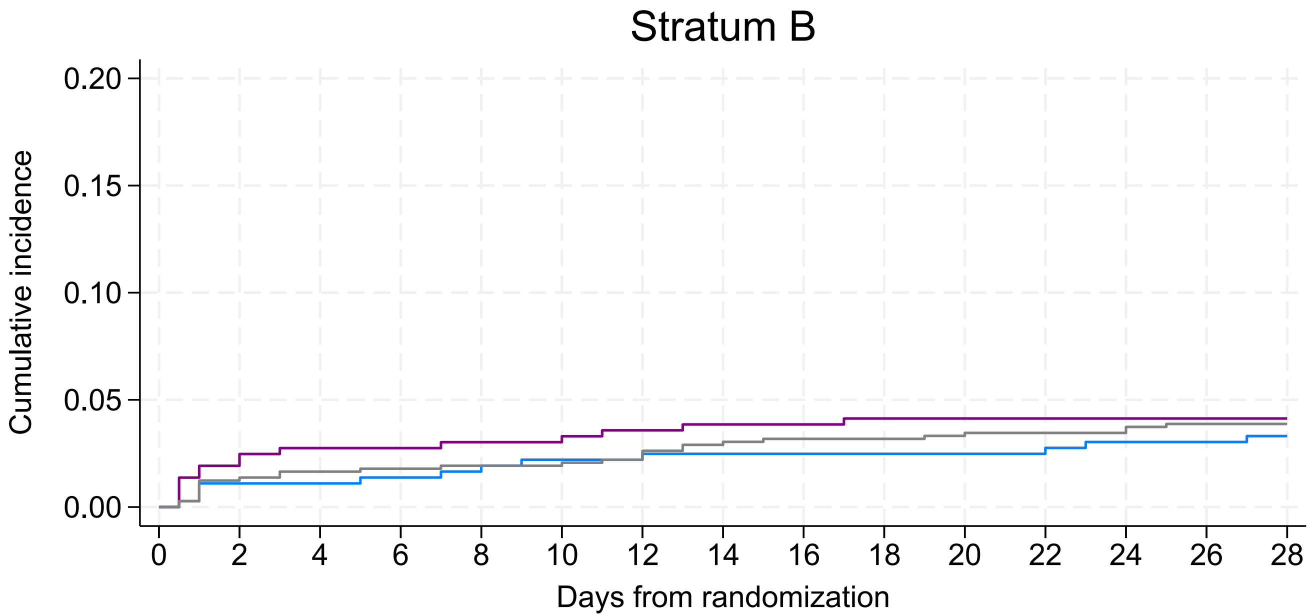

Number at risk

	0	2	4	6	8	10	12	14	16	18	20	22	24	26	28
High flow	363	359	359	357	356	354	354	353	353	353	353	353	351	351	350
Low flow	364	356	353	353	351	351	349	348	348	347	347	347	347	347	347
Control	727	716	712	708	706	705	703	698	696	696	694	693	693	690	690

Figure S4 Kaplan Meier Curves for Death to Day 28
